# Excessive Unbalanced Meat Consumption in the First Year of Life Increases Asthma Risk in the PASTURE and LUKAS2 Birth Cohorts

**DOI:** 10.3389/fimmu.2021.651709

**Published:** 2021-04-27

**Authors:** Alexander J. Hose, Giulia Pagani, Anne M. Karvonen, Pirkka V. Kirjavainen, Caroline Roduit, Jon Genuneit, Elisabeth Schmaußer-Hechfellner, Martin Depner, Remo Frei, Roger Lauener, Josef Riedler, Bianca Schaub, Oliver Fuchs, Erika von Mutius, Amandine Divaret-Chauveau, Juha Pekkanen, Markus J. Ege

**Affiliations:** ^1^ Department of Pediatrics, Dr. von Hauner Children’s Hospital, Ludwig Maximilians University Munich, Munich, Germany; ^2^ Institute for Asthma and Allergy Prevention, Helmholtz Zentrum München, German Research Center for Environmental Health, Neuherberg, Germany; ^3^ Department of Health Security, Finnish Institute for Health and Welfare, Kuopio, Finland; ^4^ Institute of Public Health and Clinical Nutrition, University of Eastern Finland, Kuopio, Finland; ^5^ Christine Kühne Center for Allergy Research and Education (CK-CARE), Davos, Switzerland; ^6^ Department of Immunology, Children's Hospital, University of Zürich, Zürich, Switzerland; ^7^ Department of Allergology, Childrens Hospital of Eastern Switzerland, St. Gallen, Switzerland; ^8^ Institute of Epidemiology and Medical Biometry, Ulm University, Ulm, Germany; ^9^ Pediatric Epidemiology, Department of Pediatrics, Medical Faculty, Leipzig University, Leipzig, Germany; ^10^ Pediatric Pulmonology, Bern University Hospital, Department for BioMedical Research, University of Bern, Bern, Switzerland; ^11^ Department of Allergology, University of Zurich, Zurich, Switzerland; ^12^ School of Medicine, University of St Gallen, St Gallen, Switzerland; ^13^ Department of Pediatric and Adolescent Medicine, Children’s Hospital, Schwarzach, Austria; ^14^ Comprehensive Pneumology Center (CPCM), Member of the German Center for Lung Research (DZL), Munich, Germany; ^15^ Division of Paediatric Pulmonology and Allergology, Department of Paediatrics, University Children's Hospital, Inselspital, University of Bern, Bern, Switzerland; ^16^ Pediatric Allergy Department, Children’s Hospital, University Hospital of Nancy, Vandoeuvre les Nancy, France; ^17^ EA 3450 DevAH, Faculty of Medecine, University of Lorraine, Vandoeuvre les Nancy, France; ^18^ Department of Respiratory Disease, UMR/CNRS 6249 Chrono-environnement, University Hospital of Besançon, Besançon, France; ^19^ Department of Public Health, University of Helsinki, University of Helsinki, Helsinki, Finland

**Keywords:** asthma, Infancy, cow's milk, meat, introduction of solid foods, nutritional immunity, gut microbiome, latent class analysis

## Abstract

A higher diversity of food items introduced in the first year of life has been inversely related to subsequent development of asthma. In the current analysis, we applied latent class analysis (LCA) to systematically assess feeding patterns and to relate them to asthma risk at school age. PASTURE (N=1133) and LUKAS2 (N=228) are prospective birth cohort studies designed to evaluate protective and risk factors for atopic diseases, including dietary patterns. Feeding practices were reported by parents in monthly diaries between the 4^th^ and 12^th^ month of life. For 17 common food items parents indicated frequency of feeding during the last 4 weeks in 4 categories. The resulting 153 ordinal variables were entered in a LCA. The intestinal microbiome was assessed at the age of 12 months by 16S rRNA sequencing. Data on feeding practice with at least one reported time point was available in 1042 of the 1133 recruited children. Best LCA model fit was achieved by the 4-class solution. One class showed an elevated risk of asthma at age 6 as compared to the other classes (adjusted odds ratio (aOR): 8.47, 95% CI 2.52–28.56, p = 0.001) and was characterized by daily meat consumption and rare consumption of milk and yoghurt. A refined LCA restricted to meat, milk, and yoghurt confirmed the asthma risk effect of a particular class in PASTURE and independently in LUKAS2, which we thus termed unbalanced meat consumption (UMC). The effect of UMC was particularly strong for non-atopic asthma and asthma irrespectively of early bronchitis (aOR: 17.0, 95% CI 5.2–56.1, p < 0.001). UMC fostered growth of iron scavenging bacteria such as Acinetobacter (aOR: 1.28, 95% CI 1.00-1.63, p = 0.048), which was also related to asthma (aOR: 1.55, 95% CI 1.18-2.03, p = 0.001). When reconstructing bacterial metabolic pathways from 16S rRNA sequencing data, biosynthesis of siderophore group nonribosomal peptides emerged as top hit (aOR: 1.58, 95% CI 1.13-2.19, p = 0.007). By a data-driven approach we found a pattern of overly meat consumption at the expense of other protein sources to confer risk of asthma. Microbiome analysis of fecal samples pointed towards overgrowth of iron-dependent bacteria and bacterial iron metabolism as a potential explanation.

## Introduction

The development of childhood-onset asthma is certainly affected by familial factors (1). The latter involve both genes and environment, two seemingly opposing principles, often paraphrased by the paronomasia of nature and nurture. Our take on this dichotomy, however, has been revolutionized by the recent advances of microbiome research. With a strong influence on our microbiome, nutrition shapes our metagenome. In other words, what we eat essentially determines how we feed our microbial patrons and which genes we borrow from them.

This is particularly true for the first year of life, when the microbiome undergoes profound changes during the transition from breastfeeding to supplemental foods (2, 3). In the PASTURE study we have already seen that this maturation process depends on environmental stimuli and can influence the development of asthma (4). One stimulus that has been inversely related to the development of allergic diseases is the diversity of foods introduced during the first year of life (5).

In the present work, we go a step beyond diversity and explore specific feeding patterns in the first year and their relation to asthma at school age. The PASTURE birth cohort provides a unique dataset on 19 food items and the intensity of feeding from monthly over weekly to daily feeding recorded on a monthly basis. We hypothesized that specific feeding patterns exist, which depend on distinct but unknown phenomena involving different cultural backgrounds. We applied latent class analysis (LCA) to this large dataset to handle the complexity of information and to estimate patterns. To complement nurture in its literal meaning with a flavor of nature in the sense of metagenome we related feeding patterns to the gut microbiome at 12 months.

## Methods

### Study Design and Population

Both birth cohorts were set up to study the development of childhood asthma and allergies and pertinent early risk factors. PASTURE recruited 1133 children in 2002-2005 from rural areas in 5 European countries: Austria, Finland, France, Germany, and Switzerland (6). Children of mothers living on family-run livestock farms were assigned to the farm study group. The reference study group comprised children of mothers from the same rural areas but not living on a farm. The independent population-based LUKAS2 cohort was established in analogy to the Finnish arm of PASTURE. All pregnant women with scheduled delivery at Kuopio University Hospital between May 2004 and May 2005 were invited to the study without selection by area of living or occupation (7). Both studies were approved by the ethics committees of the participating institutions, and written informed consent was obtained from the children’s parents or guardians.

### Questionnaires

In both cohorts, the same questionnaires were administered at the end of pregnancy and when the children were 2, 12, 18, 24, 36, 48, 60, and 72 months of age to obtain information on frequencies of wheeze, parental atopic status, and environmental exposures with a focus on farming and nutrition (5, 8). Parents reported feeding practices by weekly and monthly diaries between month 2 and 12 with introduction of solid foods mainly from month 4 onwards. Consumption of the 19 food items (bread, cake or cookies, cereals with and without gluten, meat, fish, egg, milk, yoghurt, butter, other milk products, margarine, fruits, vegetables, nuts, chocolate, sugar and sweets, soy, cod-liver oil) was asked every four weeks in categories of at least daily, weekly, monthly or less than monthly. For most items, the questionnaire differentiated between home-made and finished products. The question on meat did not specifically focus on read meat; rather it included all sorts of meat but not fish. Asthma was defined as parental report of doctor-diagnosed asthma ever or recurrent doctor-diagnosed episodes of obstructive bronchitis between 3 and 6 years. The questionnaires were developed for the PASTURE and LUKAS2 studies based on questionnaires from the ALEX study, which were validated against a food frequency questionnaire (6, 9).

### Lung Function Measurements

In PASTURE, at the age of six years in 799 children, forced expiratory volume in 1 second (FEV1), forced vital capacity (FVC), and FEV1/ FVC-ratio was measured and z-standardized (8, 10).

### Genetics

Genotyping in the PASTURE study was performed at the Centre National de Génotypage, Evry, France, as previously described (11). For the interaction analysis, the SNPs were coded additively.

### Fecal Samples

Fecal samples were collected at the age of around 12 months and processed as described previously (4). In brief, DNA was extracted from the fecal samples at a central laboratory (THL Kuopio, Finland). For sequencing, primers F515 (5′–NNNNNNNNGTGTGCCAGCMGCCGCGGTAA–3′) and R806 (5′–GGACTACHVGGGTWTCTAAT–3′) were used to amplify the V4 region of the 16S rRNA gene (12). Demultiplexed data was imported into QIIME2-2018.661 and quality trimmed. Reads were denoised using DADA262 as implemented in QIIME2. Taxonomy was assigned to representative sequences using a naïve Bayes classifier pre-built from the 99% GreenGenes database65 specific to the 515F/806R region (13). The 16S rRNA amplicon sequence variants were used as input for prediction functional abundances using Phylogenetic Investigations of Communities by Reconstruction of Unobserved States 2 (PICRUST2) v.2.1.3-b software (14). The PICRUST2 pipeline was run with default parameters and a total of 170 KEGG pathways were inferred from the predicted KEGG ORTHOLOGY (KO) abundance table.

### Assessment of mRNA Expression at Year 1 in Peripheral Blood

Assessment of mRNA was performed as described previously (15). In brief, peripheral blood samples were collected at the age of 1 year in a PAXgene® Blood RNA tubes and frozen to -80°C within 24 hours. At the central laboratory of the Children’s Hospital of Zürich, RNA was isolated, and mRNA was reverse-transcribed into cDNA. Quantitative real-time PCR was performed on the 7900HT Fast Real-Time PCR System using the Micro fluidic card TaqMan Array system of Applied Biosystems. Data presented are normalized values for the endogenous controls (18S rRNA and beta-2-microglobulin) according to the manufacturer's instructions (Applied Biosystems).

### Statistical Analysis

Calculations were performed with SAS 9.4 (The SAS Institute, Cary, NC), MPLUS 8.2 (Muthén & Muthén), and R (R Core Team, 2019). Calculation of food introduction patterns was based on all months with at least 25% of children consuming any solid foods, resulting in the period from month 4 to 12, altogether covering 9 months. Only food items with a frequency of at least 5% over this period were considered, thereby excluding soy and cod-liver oil and thus resulting 17 of the 19 asked food items. These were used as categorical variables or dichotomized at daily, weekly, or monthly consumption, respectively. In the main analyses, we used the highest frequency of the two respective production styles, i.e., home-made and finished products, whereas in sensitivity analyses we considered them individually.

Food introduction patterns were determined by a latent class analysis (LCA) based on 4-staged ordinal variables (SAS PROC LCA Version 1.3.1 and MPLUS for cross-validation). This approach considers food introduction as a latent phenomenon, which influences the time point of introduction and frequency of consumption of any food item. The LCA computes a predefined number of latent classes (LC) and models the posterior probability of belonging to each class for each individual, thereby handling missing values of up to 8 of the 9 assessed time points per food item. Individuals are then assigned to a single LC by their highest posterior probability. The appropriate number of LCs was determined by the entropy, i.e., the sum of all highest posterior probabilities over all individuals, and the sample size adjusted Bayesian information criterion (aBIC) to render the two studies with different sample sizes comparable. If both measures did not reveal the same optimal solution, a compromise was sought considering the solution with the pronounced increment in entropy at a relatively low aBIC. The resulting LCs were labeled according to their key features where necessary. To support the identification of the key features of the classes, random forest prediction models were established (R package ranger), and variable importance was used to compare the contribution of individual variables to the prediction model.

Associations of LCA classes with potential determinants, disease outcomes and composition or functional features of the microbiota were calculated by linear or logistic/multinomial regression. Effect estimates are given with 95%-confidence intervals as adjusted odds ratios (aOR) for dichotomous outcomes and β-estimates (aß) for continuous outcomes such as lung function parameters. All regression analyses were adjusted for the study group (farm versus reference), sex, and parental history of any atopic disease in both cohorts, and in PASTURE additionally for study center and atopy. The latter was defined as a combination of the “severe” and “symptomatic” atopy phenotypes as defined previously based on measurements of allergen-specific IgE (16).

## Results

**Figure 1:**
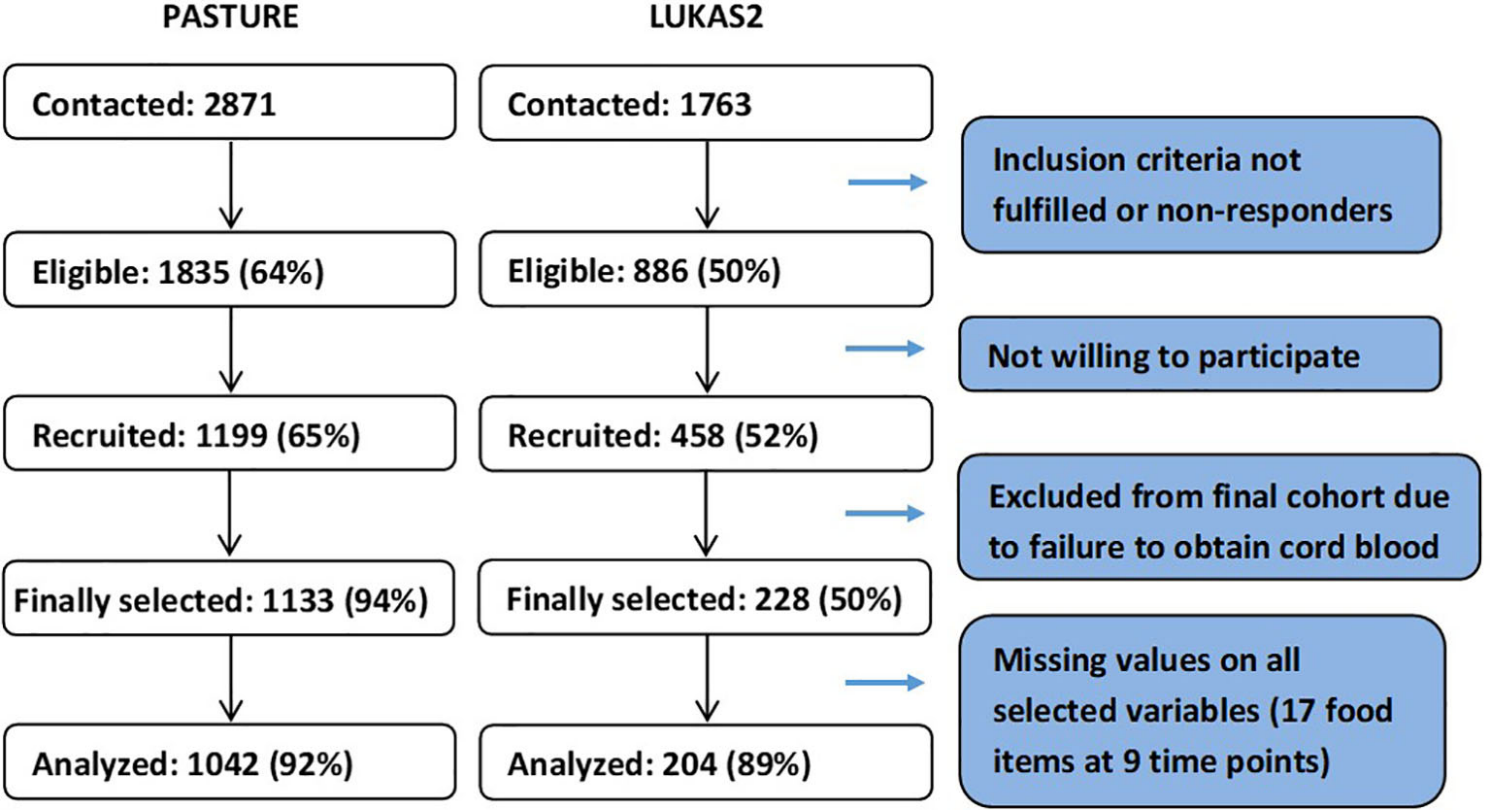
Selection of study populations.

The analysis population, as defined by available data on feeding behavior at least once per month, consisted of 1042 children (92%) from the PASTURE birth cohort (N=1133) and 204 children (89%) from the LUKAS2 cohort (N=228, **Figure 1**). Nearly 40% of the children did not have any missing values for feeding patterns, and about 90% of children had complete values for at least 50% of the variables used for calculating feeding patterns.

**Figure 2:**
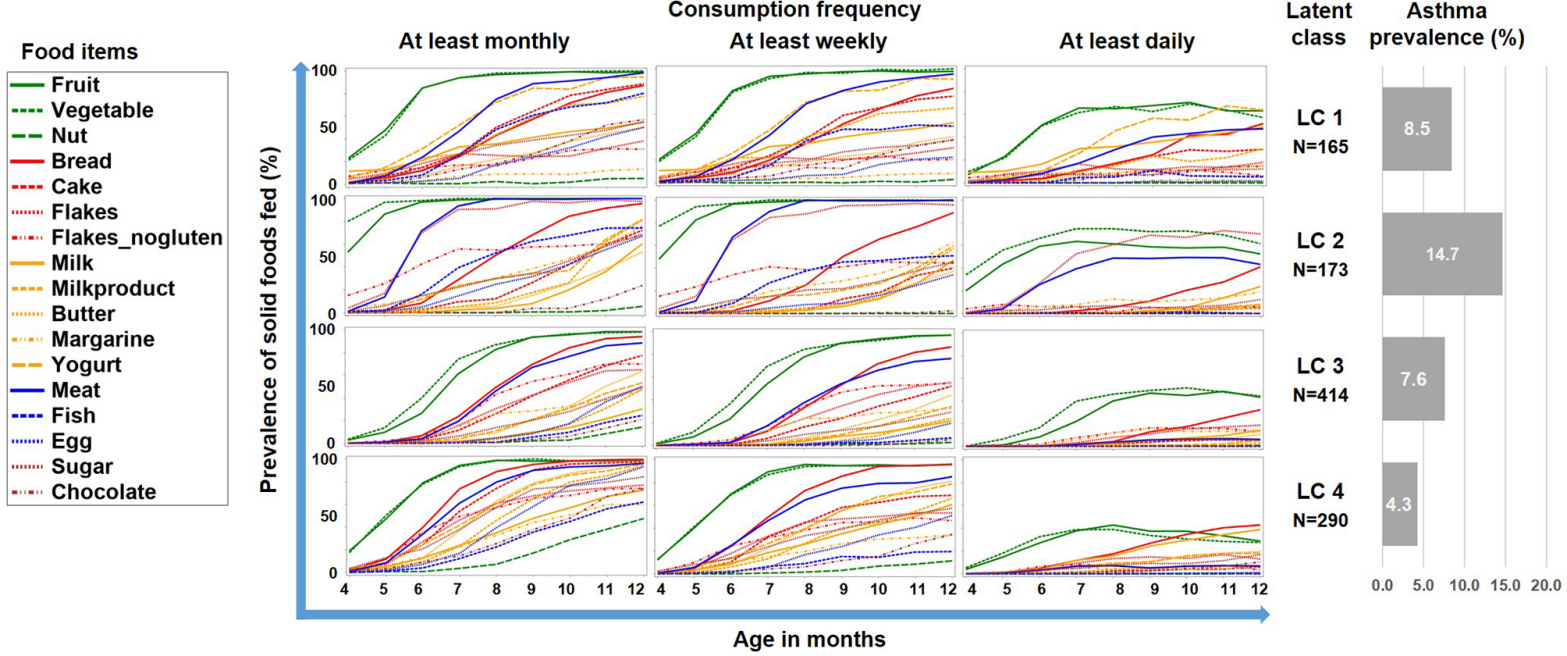
Food introduction styles in PASTURE as defined by a latent class analysis of consumption of 17 food items over 9 months.

The LCA on introduction of 17 food items at 4 frequency categories over 9 months in the PASTURE study revealed solutions with 2 to 6 classes with the 4-class solution scoring best for entropy and sample size adjusted BIC (**Table 1**). **Figure 2** illustrates the 4 resulting food introduction patterns in relation to age, frequency, and food items. The classes LC1 (n=165) and LC2 (n=173) showed a rather early introduction pattern with common daily consumption of fruits and vegetables. LC1 was additionally characterized by daily consumption of milk and milk products, which were considerably less frequent in LC2. The hallmark of LC2 was daily consumption of meat and cereals. In contrast, LC3 (n=414) and LC4 (n=290) revealed a moderate but diverse increase of food items, with daily feeding of fruits and vegetables in less than half of the infants. Monthly and weekly consumption revealed delayed introduction in LC3 as compared to LC4. The asthma prevalence in LC2 (14.7%) was much higher as compared to the other LCs (aOR for LC2 vs. the pooled other LCs: 8.47, 95% CI 2.52–28.56, p = 0.001).

**Table 1:** Model fit criteria of the latent class analyses.

Study	Food items	Number of classes	Entropy	Sample size adjusted BIC
**PASTURE**	All 17 food items	2	0.964	211771.13
		3	0.976	206688.52
		4	**0.985**	**203558.50**
		5	0.979	206923.63
		6	**0.986**	204405.74
**PASTURE**	Meat, milk, and yoghurt	2	0.921	27813.77
		3	0.909	26555.80
		4	0.916	25621.61
		5	0.924	25103.03
		6	0.924	24679.64
		7	**0.935**	24374.79
		8	0.931	**24171.74**
**LUKAS2**	Meat, milk, and yoghurt	2	0.878	4324.76
		3	0.914	4204.42
		4	0.914	**4201.71**
		5	0.953	4232.09
		6	**0.960**	4310.01

The best solutions are underlined and local extrema are marked in bold. BIC, Bayesian information criterion.

**Figure 3:**
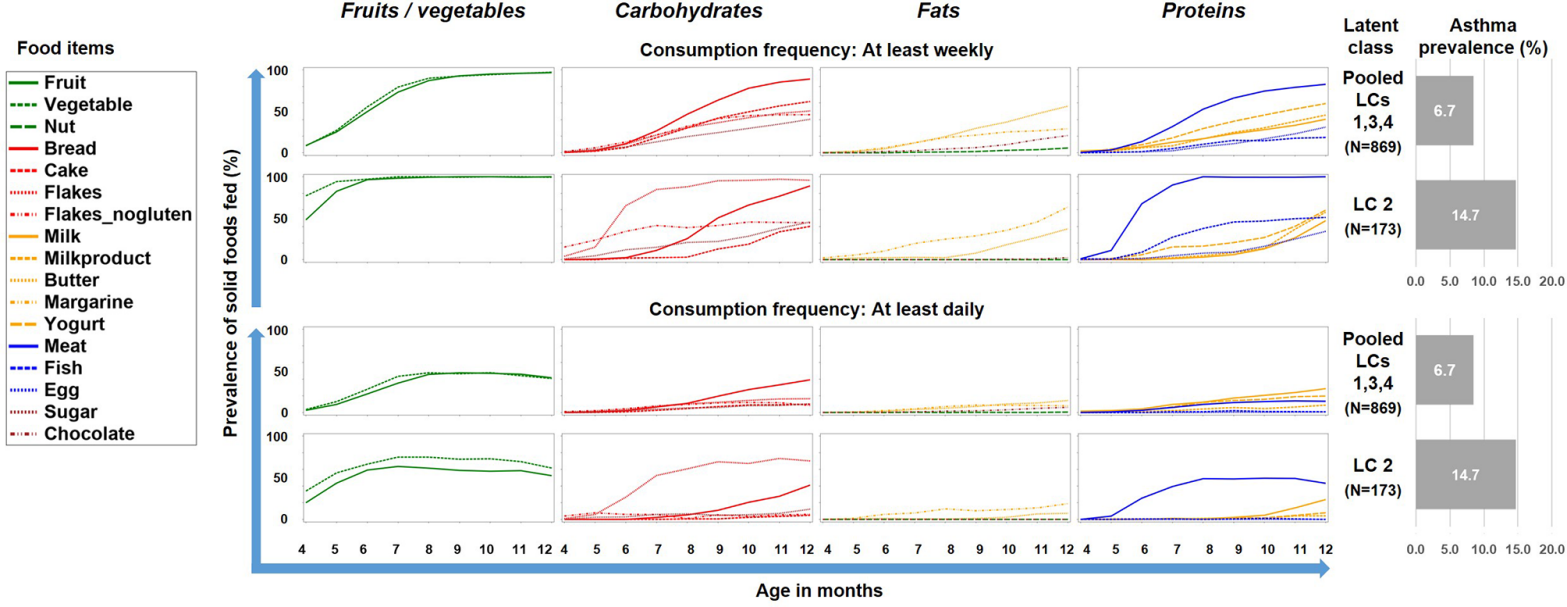
Food introduction styles stratified by macronutrients and fruits/vegetables. The asthma risk class LC2 is contrasted with a pool of the all other class (LC1, LC3, and LC4).

To better understand this phenomenon, we reduced the complexity of **Figure 2** by contrasting the asthma risk class LC2 against all other LCs and by grouping the food items by key macronutrients, i.e. carbohydrates, fat, proteins, and the group of fruits/vegetables (**Figure 3**). For fruits/vegetables and fats no relevant differences were noted, whereas among carbohydrates, consumption of cereals occurred earlier and was more common in the asthma risk class LC2. The most striking difference between LC2 and the other LCs, however, was the common daily consumption of meat with hardly any other protein source in LC2.

**Figure 4:**
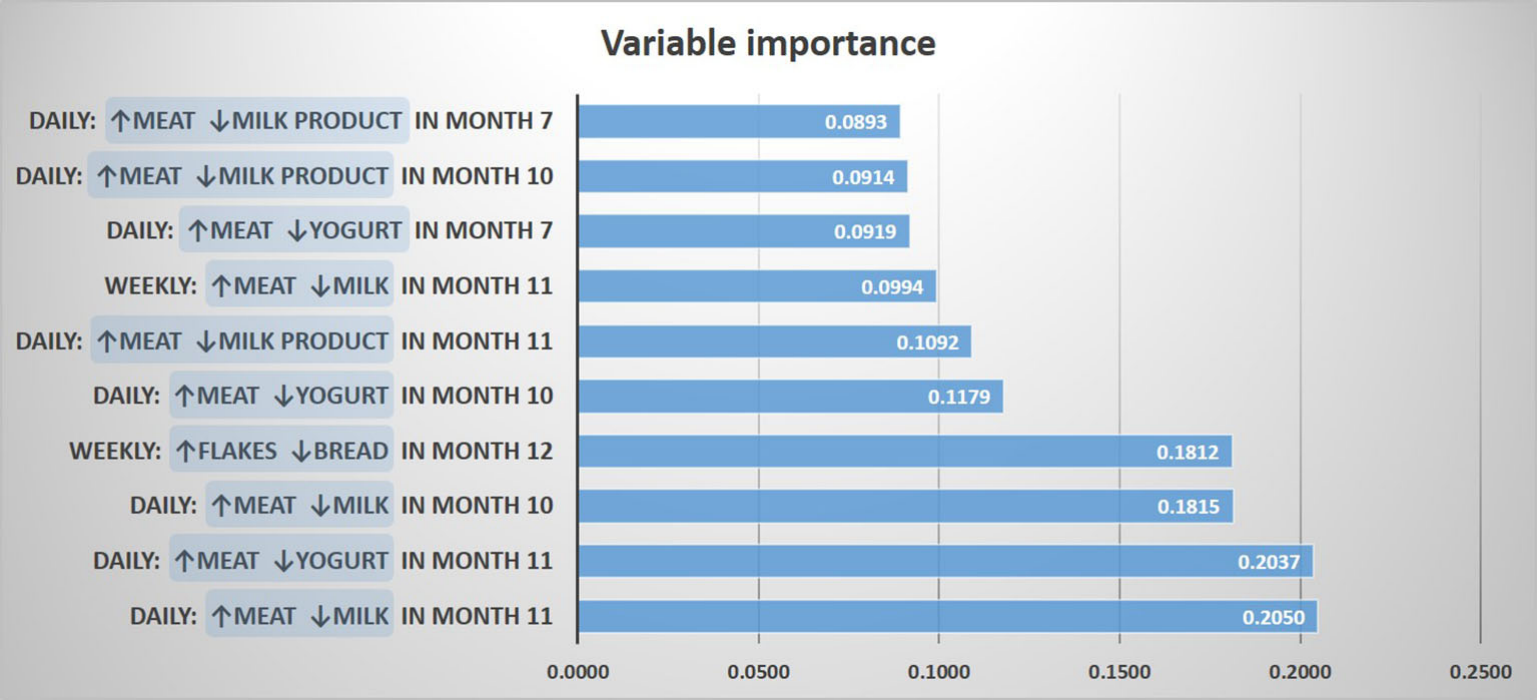
Prediction of asthma by protein and carbohydrate sources. The 10 most important prediction variables representing contrasts between food items are shown.

For determining a potential impact of these discrepancies on the asthma risk, we predicted asthma by a random forest model including contrasts between meat and all other protein sources, as well as contrasts between cereals and the other carbohydrate sources. The resulting most important variables for the prediction of asthma were “daily meat but no daily milk intake in month 11”, and “daily meat but no daily yogurt intake in month 11”, followed by variables mostly dealing with meat and milk products between month 7 and 11 (**Figure 4**). In a logistic regression model, the variables “daily meat but no daily milk intake in month 11” and “daily meat but no daily yogurt intake in month 11” were strongly associated with asthma (aOR: 2.59, 95% CI 1.31-5.13, p = 0.006 and aOR: 2.59, 95% CI 1.22-5.48, p = 0.013, respectively). On the opposite, the contrast between cereals and bread was not significantly associated with asthma (aOR: 2.04, 95% CI 0.77-5.43, p = 0.152), which led us to focus the subsequent analyses on the protein variables.

**Figure 5:**
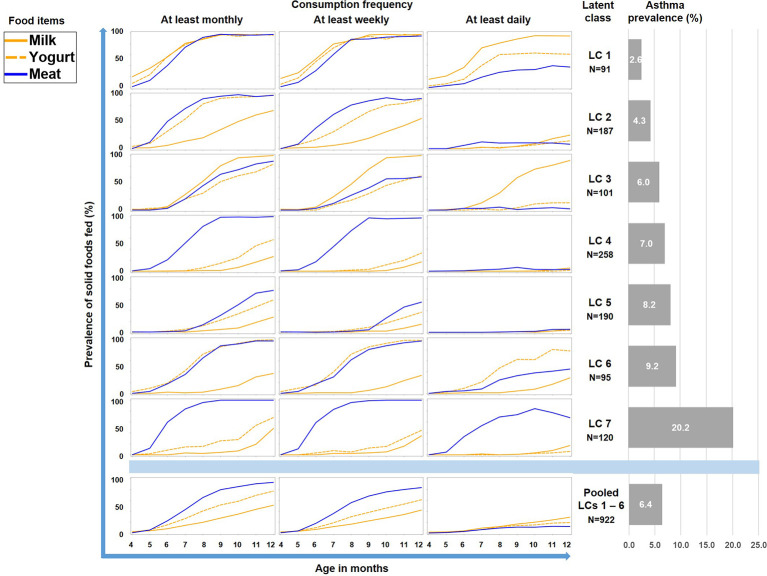
Food introduction styles in PASTURE as defined by a latent class analysis of consumption of meat, milk, and yoghurt over 9 months from month 4 to 12.

Therefore we repeated the LCA restricting food variables to the three items meat, milk, and yoghurt, which yielded a 7-class solution with the best model fit (**Table 1**). A smaller asthma risk class (LC7, n=120) emerged with a more pronounced asthma risk (20.2% asthma cases, aOR: 6.73, 95% CI 2.77-16.34, p < 0.001) as compared to all other LCs (6.4% cases, **Figure 5**). At any consumption frequency from monthly to daily, the contrast between excessive meat and low milk / yoghurt consumption was obvious in the asthma risk class LC7. We therefore termed LC7 “unbalanced meat consumption” (UMC). The pool of all other LCs showed a rather balanced pattern of meat and milk / yoghurt intake (**Figure 5**). The hallmark of the class with the lowest asthma risk (LC1, 2.6%) was less frequent meat consumption as compared to milk consumption (aOR: 1.28, 95% CI 1.18-2.03, p = 0.001).

**Figure 6:**
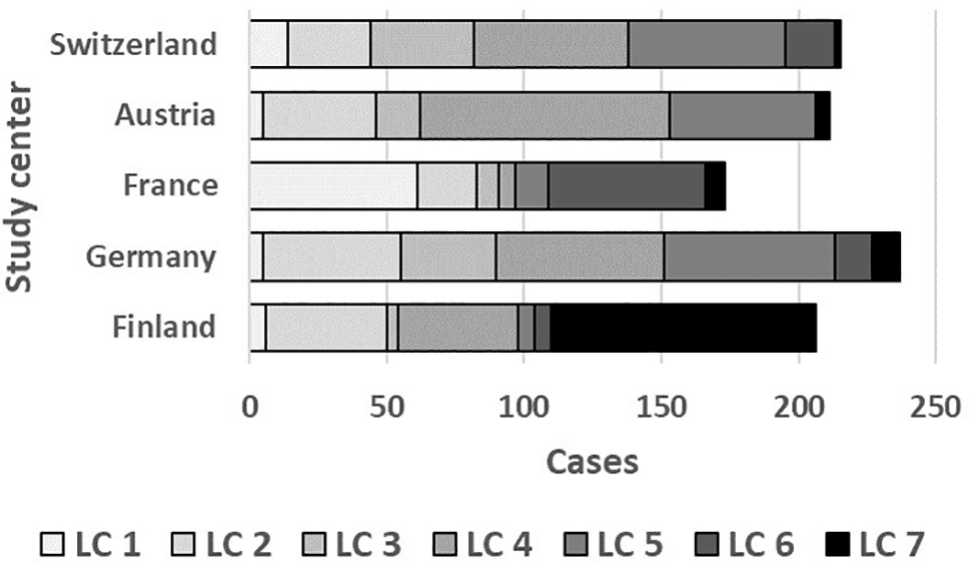
Distribution of latent classes across study centers in PASTURE.

**Figure 7:**
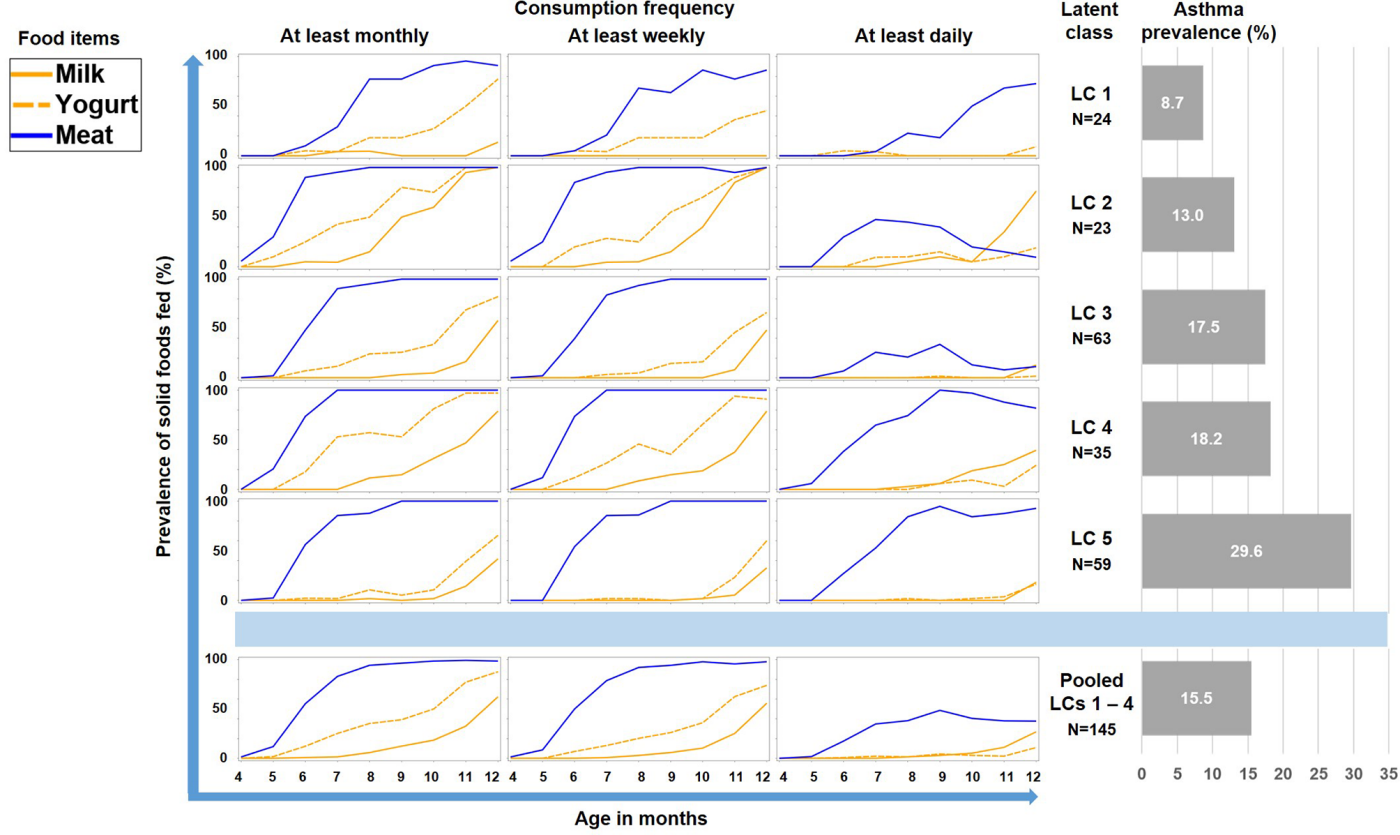
Food introduction styles in LUKAS2 as defined by a latent class analysis of consumption of meat, milk, and yoghurt over 9 months.

As illustrated by **Figure 6**, LC7/UMC was most common in Finland though it was present in all study centers. The effect of UMC on asthma in the entire PASTURE population (aOR: 6.73, 95% CI 2.77–16.34, p < 0.001) was also present within the Finnish arm (aOR: 13.42, 95% CI 3.17–56.89, p < 0.001), and with borderline significance in the other centers (aOR: 3.99, 95% CI 0.96–16.63, p = 0.057). To exclude residual confounding by center, we replicated the LCA in a purely Finnish cohort, i.e. LUKAS2. In this smaller sample, the 5-class solution provided the best compromise of entropy and sample size adjusted BIC (**Table 1**). The LUKAS2 LCA identified a class (LC5) with a similar pattern of unbalanced meat consumption and elevated asthma risk (aOR=2.32, 95% CI 1.06–5.08, p = 0.035, **Figure 7**), thereby replicating the findings from PASTURE.

**Figure 8:**
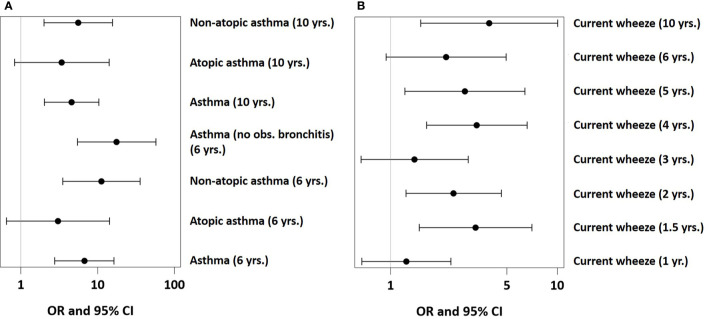
Effects of UMC on asthma (panel **A**) and wheeze (panel **B**) phenotypes in PASTURE.

In PASTURE, the effect of UMC on asthma at age 6 (aOR=6.73, 95% CI 2.77–16.34, P < 0.001) persisted until age 10 (aOR=4.35, 95% CI 1.91–9.93, P < 0.001, **Figure 8A**). The effect on atopic asthma was much weaker as compared to non-atopic asthma. When defining an asthma diagnosis irrespectively of recurrent bronchitis, the risk effect was stronger (aOR=17.0, 95% CI 5.2–56.1, p < 0.001). UMC was also significantly associated with current wheeze until age 10 with few exceptions (**Figure 8B**). For wheeze at month 18, we found significant associations of UMC only in the asthma risk strata of the single-nucleotide polymorphisms at the 17q21 locus related to ORMDL3 (rs8076131) and GSDMB (rs7216389 and rs2290400, all p-values for interaction < 0.001).

Furthermore, UMC was associated with parameters indicating poor lung function at age 6 such as FEV1 (aβ=-0.49, 95% CI -0.82; -0.16, p=0.0040) and FVC (aβ=-0.54, 95% CI -0.88;-0.19, p = 0.0023).

**Figure 9:**
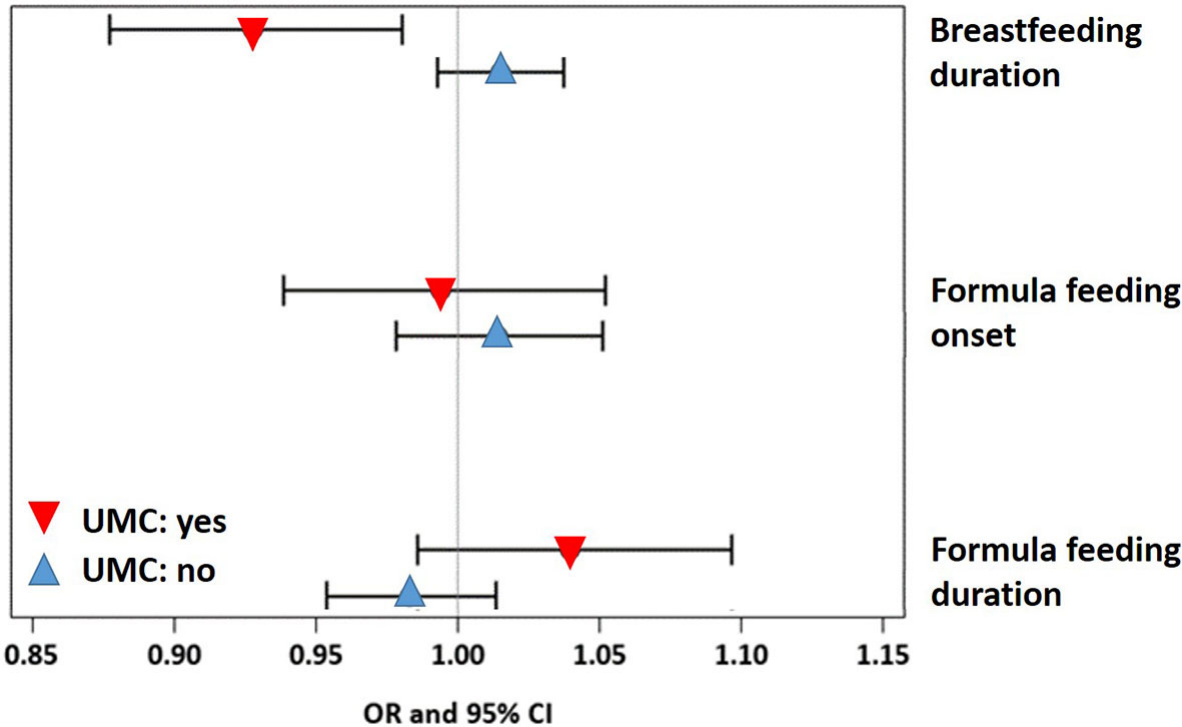
Associations of asthma with breastfeeding and formula feeding stratified by UMC.

A sensitivity analysis demonstrated that the effect of UMC on asthma (aOR=7.57, 95% CI 3.03-18.93, p < 0.001) did not change when adjusting for duration of breastfeeding (aOR=7.58, 95% CI 3.03-18.98, p < 0.001) or formula feeding (aOR=7.62, 95% CI 3.04-19.09, p < 0.001). UMC, however, significantly interacted with the effects of duration of breastfeeding and formula feeding on asthma (**Figure 9**). In children with shorter breastfeeding or prolonged formula feeding , UMC increased the risk of asthma, impairment of lung function, and weight gain until year 2 (**Table 2**). Furthermore, UMC was related to a biomarker of intestinal inflammation: CD40L expression was increased in children with UMC, particularly when duration of breastfeeding was shorter (aβ=0.89, 95% CI 0.14-1.64, p = 0.0198, **Table 2**).

**Table 2:** Effects of UMC stratified by duration of formula feeding and breastfeeding.

	All children	Formula feeding up to 28 weeks of life	Formula feeding more than 28 weeks of life	Breastfeeding up to 19 weeks of life	Breastfeeding more than 19 weeks of life
**Asthma 6 years**	aOR=7.57 [3.03-18.93] **p< 0.0001**	aOR=5.34 [0.89-32.01] p= 0.0666	aOR=12.1 [3.9-37.8] **p< 0.0001**	aOR=11.61 [3.95-34.17] **p< 0.0001**	aOR=4.14 [0.66- 26.19] p= 0.1309
**FEV1 z-score**	aβ=-0.54 [-0.89; -0.19] **p= 0.0025**	aβ=-0.35 [-1.02; - 0.32] p= 0.3000	aβ=-0.65 [-1.06; -0.25] **p= 0.0015**	aβ=-0.79 [-1.25; -0.34] **p= 0.0007**	aβ=-0.20 [-0.75; +0.35] p= 0.4666
**Weight change (g) birth – 2 years**	aβ=286 [-116; +689] p= 0.1627	aβ=-207 [-867; -452] p= 0.5375	aβ=+504 [-6; +1015] p= 0.0527	aβ=+678 [+144; +1212] **p= 0.0128**	aβ=-257 [-877; +363] p= 0.4160
**mRNA expression CD40L 1 year**	aβ=+0.43 [-0.06; +0.93] p= 0.0847	aβ=+0.11 [-0.51; +0.72] p= 0.7329	aβ=+0.65 [-0.07; +1.37] p= 0.0776	aβ=+0.89 [+0.14; +1.64] **p= 0.0198**	aβ=-0.15 [-0.73; +0.43] p= 0.6020

Duration of formula feeding and breastfeeding was dichotomized at the cut-off where the interaction for the effect on asthma was maximized. Significant associations are printed in bold. All effects were calculated in the subset of children with data for formula feeding weeks (N=972) and breastfeeding weeks (N=1069).

**Figure 10:**
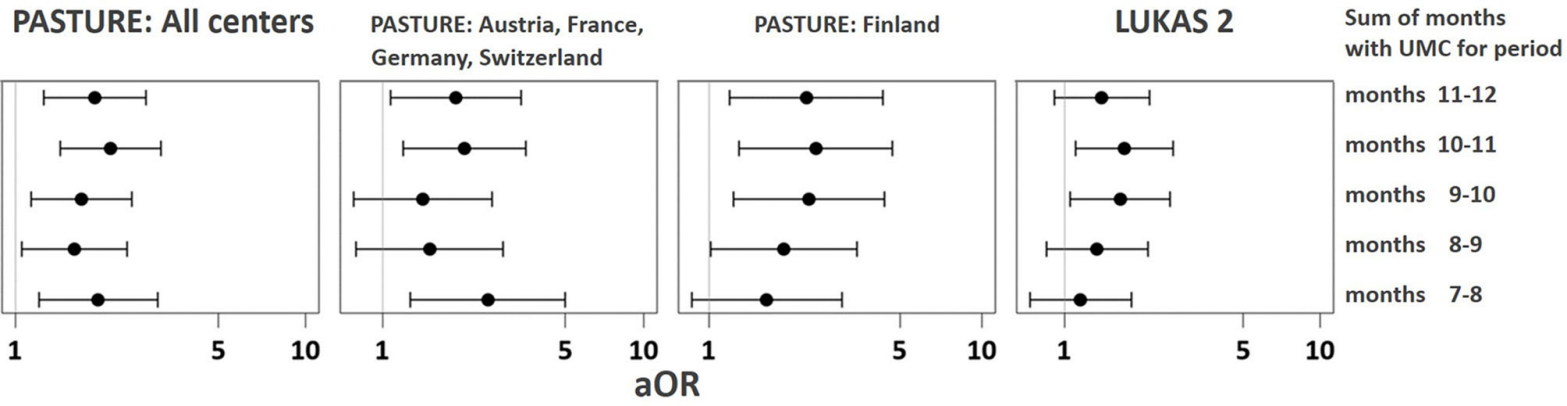
Vulnerable window for the effect of UMC on asthma at age 6. UMC is coded as daily meat consumption without milk or yoghurt consumption at least on a weekly level.

**Figure 11:**
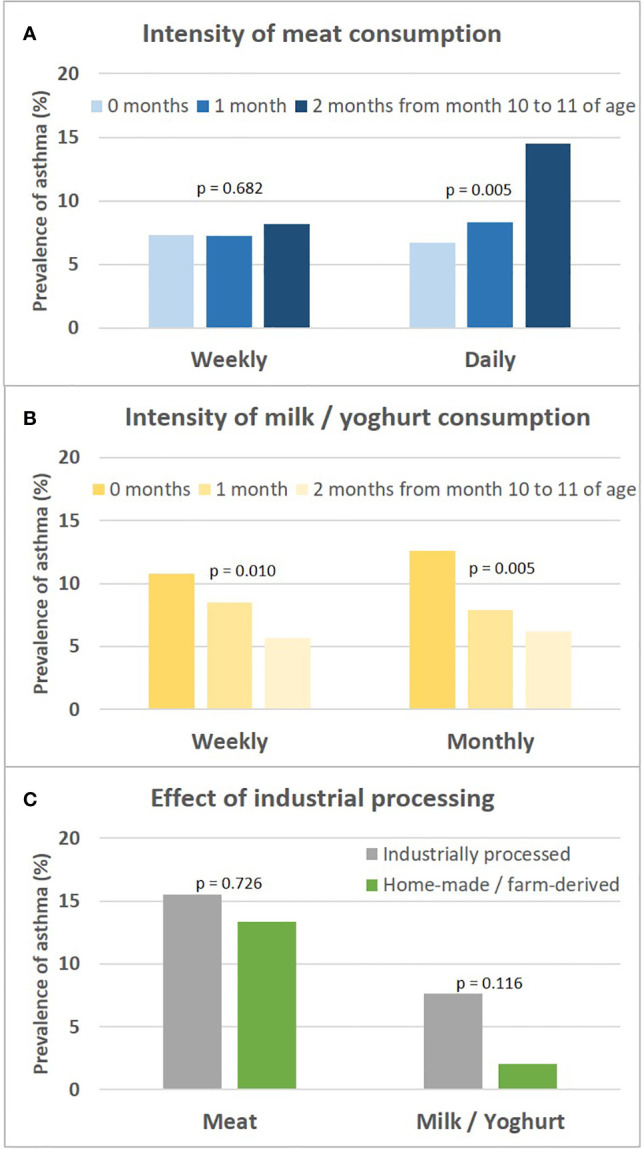
Sensitivity analyses on intensity of meat and milk or yoghurt consumption and the role of industrial processing. Shown is the prevalence of asthma in relation to the intensity of meat and milk/yoghurt consumption during month 10 and 11 (**A, B**, respectively). **(C)** presents prevalence of asthma in relation to industrial food processing; for meat, excessive consumption (daily over both months 10 and 11) and for milk, moderate consumption (weekly either during month 10 or 11) is displayed. For bivariate comparisons, we used the Chi-square or Fisher’s exact test, for trends we used the Cochran-Armitage trend test.

The asthma risk effect was particularly strong with UMC at 10 to 11 months of age (**Figure 10**). When zooming in on this vulnerable window, the effect showed a threshold phenomenon with a risk only at daily meat consumption over both months, whereas the beneficial counterbalancing effect of milk consumption tended to increase continuously with amount of consumption (**Figures 11A, B**, respectively). The effect of UMC was hardly related to industrial processing of meat (finished products versus homemade meat meals), whereas for farm milk the protective effect was stronger as compared to industrially processed milk (**Figure 11C**).

**Figure 12:**
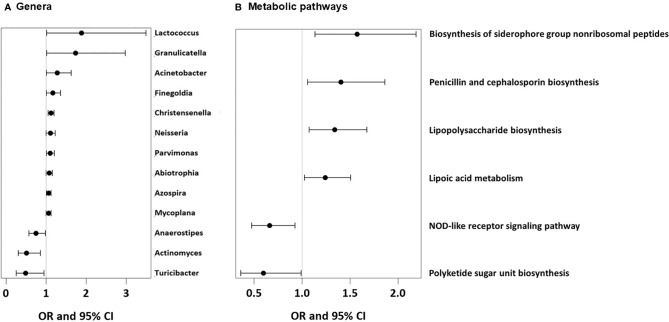
Microbial genera and microbial metabolic pathways associated with UMC. **(A)** Odds ratios (OR) with 95%-confidence intervals for the associations of UMC with relative abundance of bacterial genera in fecal samples. **(B)** Odds ratios (OR) with 95%-confidence intervals for the associations of UMC with relative abundance of metabolic pathways in fecal samples.

As we could not measure direct effects of dietary patterns on the gut physiology, we used the available gut microbiome data as a proxy for any impact of the feeding patterns. UMC influenced the composition of the gut microbiome at 12 months and fostered genera such as Lactococcus (aOR=1.88, 95% CI 1.01-3.5, p = 0.046), Granulicatella (aOR=1.74, 95% CI 1.01-2.98, p = 0.045), and Acinetobacter (aOR=1.28, 95% CI 1.00-1.63, p = 0.048, **Figure 12A**), of whom the latter genus was also related to asthma (aOR=1.55, 95% CI 1.18-2.03, p = 0.001). Most of the Acinetobacter amplicon sequence variants (ASVs) were compatible with the species johnsonii and calcoaceticus / pittii (**Table 3**). For the ASV compatible with calcoaceticus / pittii, the association was particularly strong at borderline significance (aOR=2.62, 95% CI 0.93-7.39, p = 0.068). To explore potential influences of dietary patterns on functional properties of the microbiota, we explored metabolic pathways of the microbiota and their relations to UMC. Biosynthesis of siderophore group nonribosomal peptides showed the strongest association with UMC (aOR=1.58, 95% CI 1.13-2.19, p = 0.007, **Figure 12B**).

**Table 3:** Sequences of the genus Acinetobacter.

Amplicon sequence variants (ASVs)	Reads	Species	Identity	Identity with A. baumannii
c6357b5b5bb8c067c51faad5180b4e99	402	johnsonii	99.6%	98.0%
50a3b56e0b7db75ee9daccf7a751ba41	339	johnsonii	100%	97.6%
588b5ccdde9b0d39d61568731d7e223f	173	calcoaceticus, lactucae, nosocomialis, pittii, oleivorans	100%	98.0%
107aa7e6b56274803bffd1ccff5b2ee6	150	colistiniresistens, gerneri, proteolyticus, courvalinii, wuhouensis, gyllenbergii, junii, tandoii, guillouiae, bereziniae Nakamurella silvestris	100%	96.8%
2f41b4b15416f046360ecaf1cfeb4b28	24	schindlerijohnsonii	100%99.6%	
5c96ec70d2839b27eb48f2226c96e2ca	16	idrijaensis, pseudolwoffii, lwoffii	100%	
262dc7bf78e937b0c7065aef82e18d08	13	parvus, tjernbergiae, beijerinckii,disperses, haemolyticus	100%	

## Discussion

By systematically assessing feeding patterns in the first year of life we discovered a feeding type associated with high risk of asthma at school age. We termed the pattern ‘unbalanced meat consumption’ (UMC) because daily intake of meat was by far the predominant protein source. This pattern was rather common in Finnish children; the association with asthma, however, was independent of center in the multicenter PASTURE birth cohort and found similarly within the independent Finnish LUKAS2 population. Prolonged formula feeding potentiated the detrimental effect of UMC on asthma, lung function, weight gain, and induced inflammation as measured by expression of CD40L. UMC boosted growth of *Acinetobacter* and *Christensenella*, which have previously been found asthma risk genera (4, 17). In addition, UMC fostered growth of bacteria with the capability to synthesize siderophore peptides.

Latent class analysis (LCA) offers a data-driven approach to unveil latent factors behind measurable variables (18). Therefore, we used this approach to perform an unbiased analysis of the complex phenomenon of introduction of solid foods during the first year of life. Though the diaries did not provide information on serving size or the precise weight of foods, they covered information on frequency of feeding from monthly over weekly to daily. This information was particularly helpful as it allowed differentiation of feeding patterns by intensity and ultimately revealed the asthma risk class with daily meat consumption. Furthermore, the usage of LCA discovered specific combinations of foods such as the balanced combination of meat and milk / yoghurt consumption (LC1 in **Figure 5**) with its low asthma risk. This created a strong contrast to the asthma risk class (UMC, i.e. LC7 in **Figure 5**), in which consumption of milk or yoghurt was very rare. We considered the strong preference of meat over other protein sources an obvious misbalance and thus termed the risk phenomenon ‘unbalanced meat consumption’ (UMC).

Though being aware of the disease relevance of the diversity of foods introduced during the first year (5), we were surprised by the clear-cut risk class emerging from the primary LCA. To understand better this phenomenon we first stratified the 17 food items by macronutrients and found protein and carbohydrate sources to be relevant. When predicting asthma directly from the food items using a random forest model, we realized that the characteristic intensive consumption of flakes in the asthma risk group was only an epiphenomenon to the essential contrast between meat and milk / yoghurt consumption. Milk products other than yoghurt (e.g. butter) seemed to be less important.

Under the assumption that other food items might have increased statistical noise, we refined the LCA by restricting the food items entered in the model to meat, milk, and yoghurt. This advanced LCA sharpened the contrast between meat and milk / yoghurt consumption and its effect on asthma. Since the selection of entry variables was based on the previous random forest for asthma, the stronger association was expected and possibly an effect of circular reasoning. Therefore, we replicated this finding in an independent population. The latter consisted only of Finnish children, which at the same time excluded confounding by center, country, or possibly by genetic background.

The effect of UMC in the first year was even detectable at 10 years, when asthma risk was still positively associated with UMC. Consequently, we assume that the feeding pattern resulted in fundamental, potentially epigenetic, changes persisting over at least 10 years and impacting on objective lung function parameters. Since the effect was stronger on non-atopic asthma, an allergic component in the pathomechanism seems less probable. The stronger effect on asthma irrespectively of early recurrent bronchitis also renders involvement of viral infections in the pathogenesis unlikely. Though UMC was also associated with early wheeze, this association was restricted to the asthma risk genotype. Early wheeze in individuals with genetic asthma risk encoded on chromosome 17q21 is highly suggestive of subsequent asthma rather than just transient viral wheeze (19).

To better characterize UMC we performed various sensitivity analyses and found the effect of UMC particularly pronounced at 10 to 11 months of age thereby suggesting a susceptible window. Moreover, the asthma risk was only present when daily consumption was maintained over both month 10 and 11 supporting the notion that quantity and duration of unbalanced meat consumption matters. Whereas for milk and yoghurt the well-known effect of unprocessed milk on asthma was detected (9), there was hardly any influence of industrial processing on the effect of UMC, pointing towards meat itself as the culprit irrespectively of artificial additives. However, we have not asked for the processing status of meat in full detail, thus we might have missed a detrimental effect by industrially processing as described for meat consumption and impaired lung function in adults (20).

Since concomitant milk consumption seemed to abrogate the detrimental effects of excessive meat, we explored also other types of milk that were excluded from the LCA of supplemental foods by definition, i.e. breast milk and formula milk. We found short duration of breastfeeding and prolonged formula feeding to enhance the effects of UMC. Initiation of formula feeding may be a proxy for duration of breastfeeding in the PASTURE study (21); alternatively, duration of formula feeding may point towards an independent source of milk and thereby to delayed introduction of normal cow’s milk and yoghurt. In contrast, breastfeeding may counterbalance the detrimental effects of excessive meat similarly to (mildly processed) cow’s milk.

The interaction of UMC with breastfeeding for the expression of CD40L, a marker of both allergic and intestinal inflammation (22), is highly interesting. In the absence of the anti-inflammatory properties of maternal milk (23, 24), UMC might induce (low-grade) inflammation of the intestinal wall possibly leading to systemic T-cell activation (22). Admittedly, the evidence provided by the data available now is weak but the postulated link between feeding patterns and intestinal inflammation might be a promising direction for future research.

Beyond local mucosal inflammation, the question remains how UMC may influence a rather distant organ system and affect the pathogenesis of asthma. Because we have previously seen an effect of consumption of milk and eggs on the gut microbiome at 12 months and its effect on asthma (4), we again followed this route. The particularly pronounced effects of UMC at 10 to 11 months rendered an assessment of the gut microbiome at 12 months an ideal situation. Given the exploratory approach, the associations with microbiota were not corrected for multiple testing. With this caveat in mind, we found several taxa to be possibly fostered by UMC. By a Picrust analysis of functional properties, the common denominator of these taxa was unraveled: The top hit of the biosynthesis pathways pointed towards siderophore group nonribosomal proteins and thus to bacterial iron metabolism. This notion is supported by a study on iron supplementation in pregnant women, which revealed an association of high supplementation with the very same pathway in the gut microbiome (25). Siderophores are employed by intestinal bacteria in their battle for nutritional iron. More than 80% of the dietary iron is not resorbed and passed to the colon, where adverse gut bacteria may overgrow and lead to inflammation (26). At a closer look, most of the genera associated with UMC were actually well-known iron scavengers or even pirates. This is particularly documented for *Neisseria*, a known pathogen (27). Likewise, *Acinetobacter baumanii* is a human pathogen closely related to the here identified species *calcoaceticus / pitti* (28). The mentioned *Acinetobacter* species form together the phenotypically homogenous *Acinetobacter calcoaceticus–Acinetobacter baumannii* complex (29), which plays a role in airway infections such as pneumonia (30, 31).

As a countermeasure, the host organism can launch a number of defense mechanisms and establish “nutritional immunity” by limiting bacterial acquisition of necessary nutrients such as metals (32, 33). Bacteria can be kept at bay by the innate bactericide function of lipocalins (34), which are secreted by gut epithelial cells and possess the ability to neutralize siderophores (35). In addition, cow’s milk contains a molecule of the lipocalin family: β-lactoglobulin. This molecule has previously been found at high levels in unprocessed cow’s milk and has been directly related to lower asthma risk (36). Likewise, lactoferrin, another defense molecule secreted to the gut lumen and to milk, can sequester siderophores (37, 38). Remarkably, both molecules emerged as candidates for the beneficial effect of farm milk, when not inactivated by high heat treatment (9, 36, 39). Since formula milk is produced from ultra-heat treated milk, these molecules are no longer active, which might explain the detected potentiation of the unfavorable UMC effect by prolonged formula feeding. In contrast, the intact compounds of breastmilk might have alleviated the disadvantages of UMC.

We have previously postulated a gut-lung axis in humans mediated by maturation of the gut microbiome with involvement of short chain fatty acids (4). Though associated with some asthma risk bacteria including *Acinetobacter* (4) and *Christensenella* (17), UMC exerted an effect on asthma independently from gut maturation (data not shown), thereby suggesting a second component to the gut-lung axis. A parallel finding might be seen in a recent gut metabolome study suggesting consumption of processed meat and lack of breastfeeding to induce a metabolic profile associated with subsequent development of asthma (17).

The above mentioned mechanisms of nutritional immunity, which may be compromised by UMC, may affect the entire organism since competition for iron and other metals is a systemic effect as illustrated by the well-known phenomenon of infectious anemia (40). Interestingly, the airway pathogens *Moraxella catharrhalis* and *Haemophilus influenzae*, which are specifically associated with wheeze and asthma (41, 42), are known to command potent strategies of iron acquisition involving lactoferrin and transferrin binding proteins (33).

The opposite health effects of milk consumption and excessive meat intake may be explained by a number of other known biological mechanisms, such as discrepant fatty acid patterns (43, 44), different impact on oxidative stress (45), or involvement of non-microbial, non-human antigens such as Neu5Gc (46), and Alpha-Gal (47), which are related to milk and red meat and influence human health in different directions. In the present analysis, the UMC finding could not be attributed to the effects of fatty acid patterns, Neu5Gc and Alpha-Gal (data not shown), and measurements of oxidative stress were not available in our cohorts. Besides, the interaction itself would not be explained by the above-mentioned alternatives.

The interplay between nutritional iron and overgrowth of potentially adverse bacteria on one side and defense mechanisms possibly supported by dietary milk-derived proteins on the other side may eventually explain the detected interaction of meat and milk intake (UMC) for asthma. This would provide a coherent and simple rationale compatible with the principle of simplicity, which is inherently more attractive (48). Future, more sophisticated studies may prove the hypothesized mechanism on a molecular level, while currently we only describe statistical associations. These limitations might be compensated by the specific strengths of this analysis, i.e. the replication in two independent cohorts, the detailed nutritional diary, and the long follow-up of children until school age.

Whether infants, particularly in Finland, are nowadays fed on excessive meat may be doubted, given the rapidly evolving evidence-based adjustments to Public Health measures (https://thl.fi/en/web/handbook-for-child-welfare-clinics) (49). If corroborated by other studies, it will be advisable to avoid excessive daily meat intake at home and in day care facilities, particularly during the vulnerable window of introduction of solid foods. Rather the consumption of mildly processed cow’s milk may contribute to a healthy diet beyond the already known beneficial effects (9).

Beyond the Public Health dimensions, the here presented finding might be important for a deeper understanding of asthma pathogenesis. The postulated effect of microbial imbalance towards iron scavenging gut bacteria may result in long−term immune dysfunction and low−grade inflammation as involved in asthma, obesity, inflammatory bowel disease, and adverse metabolic conditions (49).

Taken together, we performed an unbiased analysis of feeding patterns in the first year of life and discovered a constellation of excessive meat consumption at the expense of other protein sources, which was characterized by a substantially elevated asthma risk. With the help of microbiome analyses we identified bacterial iron metabolism as a likely culprit for the adverse health phenomenon. Though not revealing final proof for this hypothesis, the current work may stimulate research into the effects of nutritional iron and other metals on the development of asthma and lead to novel preventive approaches.

## Data Availability Statement

PASTURE and LUKAS2 are two ongoing birth cohorts with fieldwork still being executed and biosamples not yet used up. As long as the studies are not yet anonymized and as long as the biosamples are not used up, European data protection legislation prohibits sharing of individual data (also when pseudonymized) to guarantee participant privacy.

## Ethics Statement

The studies involving human participants were reviewed and approved by Local ethics committees. Written informed consent to participate in this study was provided by the participants’ legal guardian/next of kin.

## PASTURE Study Group Members

Anne Hyvärinen, PhD,^3^ Sabina Illi, PhD,^1^ Michael Kabesch, MD,^16^ Lucie Laurent, PhD,^13^ Petra I. Pfefferle, PhD,^18,24^ Harald Renz, MD, PhD,^17,18^ Marjut Roponen, PhD,^15^ Urs Frey, MD, PhD,^25^

^1^ Institute for Asthma and Allergy Prevention, Helmholtz Zentrum München, German Research Center for Environmental Health, Neuherberg, Germany; ^3^ Department of Health Security, Finnish Institute for Health and Welfare, Kuopio, Finland; ^13^ Department of Respiratory Disease, UMR/CNRS 6249 Chrono-environnement, University Hospital of Besançon, France; ^15^ Department of Environmental and Biological Sciences, University of Eastern Finland, Kuopio, Finland; ^16^ KUNO Children’s University Hospital Regensburg, Department of Pediatric Pneumology and Allergy, Regensburg, Germany; ^17^ Department of Clinical Chemistry and Molecular Diagnostics, Philipps University of Marburg, Marburg, Germany; ^18^ Member of the German Center for Lung Research; ^24^ Comprehensive Biomaterial Bank Marburg CBBM, Philipps University of Marburg, Marburg, Germany. ^25^ University Children’s Hospital Basel (UKBB), University of Basel, Basel, Switzerland.

## Author Contributions

EvM, JR, and JP obtained funds, set up the PASTURE birth cohort and had responsibility for data collection and management of the study. CR, RF, RL, PK, AK, AC, BS, and OF were involved in acquisition, and interpretation of data. JG and EH were involved in data management. GP performed bioinformatics. AH and MD performed statistical analyses; ME supervised statistical analyses. AH and ME drafted the manuscript. All authors contributed to the article and approved the submitted version. The PASTURE study group were involved in acquisition, management and interpretation of data in Austria, Finland, France, Germany, and Switzerland.

## Funding

The PASTURE birth cohort has been supported by the European Commission (research grants QLK4-CT-2001-00250, FOOD-CT-2006-31708, and KBBE-2007-2-2-06), the European Research Council (Grant 250268). Biosamples were stored at the Comprehensive Biobank Marburg CBBMR, Philipps University of Marburg, Marburg, Germany. The current analysis was supported by the German Center for Lung Research (DZL). The funding sources did not influence the study design; the collection, analysis, and interpretation of data; the writing of the manuscript; and the decision to submit the paper for publication.

## Conflict of Interest

EvM reports to have received personal fees from PharmaVentures, OM Pharma, Springer-Verlag, Elsevier, Peptinnovate, Turun Yliopisto, Tampereen Yliopisto, Helsingin Yliopisto, European Respiratory Society, Deutsche Pharmazeutische Gesellschaft, Massachusetts Medical Society, the Chinese University of Hong Kong, European Commission, Boehringer Ingelheim, Universiteit Utrecht Faculteit Diergeneeskunde, Universität Salzburg, Georg Thieme Verlag, Japanese Society of Pediatric Allergy and Clinical Immunology, Nestlé Deutschland and HiPP, outside of the submitted work, and has received funding and research support from FrieslandCampina. In addition, EvM has patent LU101064 (Barn dust extract for the prevention and treatment of diseases) pending, royalties paid to ProtectImmun for patent EP2361632 (Specific environmental bacteria for the protection from and/or the treatment of allergic, chronic inflammatory and/or autoimmune disorders, granted on 19 March 2014), and patents EP1411977 (Composition containing bacterial antigens used for the prophylaxis and the treatment of allergic diseases, granted on 18 April 2007), EP1637147 (Stable dust extract for allergy protection, granted on 10 December 2008), and EP 1964570 (Pharmaceutical compound to protect against allergies and inflammatory diseases, granted on 21 November 2012) licensed to ProtectImmun.

The remaining authors declare that the research was conducted in the absence of any commercial or financial relationships that could be construed as a potential conflict of interest.
